# Study on the Immune Escape Mechanism of Acute Myeloid Leukemia With DNMT3A Mutation

**DOI:** 10.3389/fimmu.2021.653030

**Published:** 2021-05-20

**Authors:** Yimei Que, Huimin Li, Liman Lin, Xiaojian Zhu, Min Xiao, Ying Wang, Li Zhu, Dengju Li

**Affiliations:** Department of Hematology, Tongji Hospital Affiliated with Tongji Medical College, Huazhong University of Science and Technology, Wuhan, China

**Keywords:** AML, DNMT3A, macrophage polarization, immune escape, immune microenvironment, epigenetics

## Abstract

DNA (cytosine-5)-methyltransferase 3A (DNMT3A)-mutated acute myeloid leukemia (AML) has a poor prognosis, but the exact mechanism is still unclear. Here, we aimed to explore the mechanism of immune escape in AML with DNMT3A mutation. We constructed a DNMT3A knockout clone and DNMT3A-R882H-mutated clones. RNA-seq results showed that transcription factors and macrophage inflammatory proteins were significantly downregulated in the DNMT3A mutant clones. KEGG enrichment and gene set enrichment analysis (GSEA) showed that a large number of genes were enriched in inflammatory immune-related pathways, such as the toll-like receptor signaling pathway. Therefore, we co-cultured AML cells with macrophages. The DNMT3A-mutated AML cells attenuated M1 macrophage polarization and resisted its killing effect *in vitro* and *in vivo*. In xenografts, the tumor volumes in the experimental group were significantly larger than those in the control group, and the proportion of M2 macrophages was significantly higher. After the co-culture, the increase in pro-inflammatory cytokine expression in the mutant cells was significantly lower than that in the control group, while that in immunosuppressive factors was not significantly different. In co-cultivated supernatants, the concentration of inflammatory factors in the experimental group was significantly lower than that in the control group, while that of immunosuppressive factors was significantly higher. Resistin significantly promoted the expression of inflammatory proteins in AML cells. It relieved the inhibitory effect of DNMT3A mutation, promoted the phenotypic recovery of the co-cultured macrophages, eliminated resistance, and regulated the immune microenvironment. Thus, resistin may serve as an ancillary drug for patients with DNMT3A-mutated AML.

## Introduction

Acute myeloid leukemia (AML) is a common, heterogeneous, and aggressive hematopoietic malignancy, characterized by apparent genetic abnormalities (1). About 25% of AML cases are accompanied by DNA (cytosine-5)-methyltransferase 3A (DNMT3A) molecular mutation. DNMT3A-mutated AML has high-risk characteristics and shows significantly worse overall survival (OS) (2), but its mechanism is unclear (3). Therefore, it is necessary to explore the underlying pathogenic mechanism and find a new treatment for DNMT3A-mutated AML.

DNMT3A is a key enzyme for *de novo* epigenetic methylation in humans. For the past several years, efforts have been made to explore functional consequences associated with the DNMT3A mutation. The DNMT3A mutation can cause significant changes in epigenetic modifications and is one of the essential regulatory factors for the occurrence and development of leukemia (4). DNMT3A mutation is an inactivating mutation. The mutated proteins can interact with normal proteins to form dysfunctional tetramers, and thus change the pattern of genome methylation (5). The degree of methylation affects the expression of target genes and has a significant impact on cell proliferation, apoptosis, cytokine secretion, and cell activity (6, 7). Some researchers believe that DNMT3A mutations cause hypomethylation of the whole genome, and this hypomethylation is often associated with a higher recurrence rate and worse OS (8). Still, other scholars speculate that hypomethylation is only the initial phenomenon of DNMT3A-mutated AML, while hypermethylation of DNMT3A-dependent CpG islands is the result of AML progression (3).

Macrophages are the most abundant white blood cells in the immune microenvironment and have a high degree of heterogeneity. They can polarize to different directions to play completely different roles in different immune microenvironments. According to the function and immune phenotype, macrophages can be divided into two types: Classically activated (M1) and alternatively activated (M2) macrophages. M1 macrophages have strong pro-inflammatory and antigen-presenting capabilities. They can inhibit the growth of tumor cells, kill them *via* phagocytosis, and promote anti-tumor immunity. M2 macrophages can inhibit inflammation, as well as promote vascular remodeling, and tumor growth and infiltration.

Communication between AML cells and the cells in the microenvironment is a two-way process that involves a wide variety of non-cancer cells and a diverse range of mechanisms (9–11).Macrophages play an essential role in the AML immune microenvironment. They can regulate the microenvironment by secreting a variety of cytokines, and bidirectionally regulate immunity through interacting with immune cells, such as T cells. Promoting the function conversion of heterogeneous immune cells, such as macrophages, regulatory T cells (Tregs) (12) are expected to eliminate AML cells further. Leukemic cells can promote immunosuppression of the microenvironment by releasing exosomes (13). The immunosuppressive state of the microenvironment can reduce the ability of macrophages to recognize and engulf antigens, and induce the M2 macrophage phenotype (14, 15). Activated M2 macrophages highly express interleukin 10 (IL-10) and transforming growth factor β (TGF-β), which can help tumors escape the host’s immune surveillance. The signals released by M2 macrophages can inhibit the production of pro-inflammatory chemokines by M1 macrophages (16). In addition, M2 macrophages can produce chemokines, such as CCL17, CCL22 and CCL24, which are related to Th2 response (17, 18), and inhibit the inflammatory response further.

The present study, therefore, aimed to elucidate the mechanism of poor prognosis of DNMT3A-mutated AML, using gene-editing from the perspective of immune escape.

## Methods

### CRISPR-Cas9-Based Gene Knockout (KO) in SKM-1 Cells

Using online tools (http://www.rgenome.net/cas-offinder/), we designed a small guide RNA (sgRNA1: 5’-GCTACCACGCCTGAGCCCGT-3’; SgRNA2: 5’-tccasgRNAccttctgagactcccc-3), which targeted *DNMT3A* exon 6. DNMT3A-targeting sgRNAs were synthesized using the HiScribe™ T7 Quick High Yield RNA Synthesis Kit (E2050S, NEB, USA) according to the manufacturer’s instructions. After being treated with 1 µg/ml doxycycline (PHR1789, Sigma, Sweden) for one week, the SKM-1 cells were stained with anti-DNMT3A antibody, and DNMT3A negative cells were sorted as DNMT3A KO cells, using TA cloning to select double-KO cell strain.

### Viral Construction and Infection

Full-length cDNA of human *DNMT3A* was cloned into the EcoRI/BamHI multiple cloning site of the lentiviral vector pCDH-EF1-MCS-T2A-copGFP. DNMT3A-R882H mutant was generated using the KAPA HiFi HotStart PCR kit (KR0369–v10.17, Kapa Biosystems, USA) and confirmed *via* full-length DNA sequencing. After 48 to 72 h, the supernatants containing infectious lentiviruses were collected, filtered through a 0.45-µM filter, and incubated with target cells for transduction. The viral titer was determined *via* flow cytometry using infected SKM-1 cells.

### TALEN and Donor Plasmids Construction

Generation of isogenic K562 clones with the DNMT3A R882H mutation using designed TALENs, as described previously (19).

### Cell culture and Cell Proliferation Assay

Human AML cells SKM-1 and THP-1 were obtained from the China Center for Type Culture Collection (Wuhan, China). All cell lines were tested and authenticated, utilizing short tandem repeat profiling every six months. SKM-1 and THP-1 cells were cultured in RPMI-1640 (GIBCO, Life Technologies Corporation, Carlsbad, CA, USA) supplemented with 10% fetal bovine serum and 100 U/mL of penicillin-streptomycin at 37°C and 5% CO_2_ and normal O_2_. The CCK8 assay (A311-01/02, Vazyme, China) was used to test the proliferation of cells, according to the manufacturer’s instructions.

### Leukemia Cells and Macrophage Co-Culture Assay

THP-1 monocytes were differentiated into macrophages *via* a 24-h incubation with 150 nM phorbol 12-myristate 13-acetate (PMA, HY-18739-1, MedChemExpress, USA). AML cells were cultured in the upper chamber of transwell inserts (HKN-3450, Corning, USA) in 6-well plates. Macrophages were placed in the lower chamber of a 6-well transwell plate (E: T; 1:1) and co-cultured for 48 h.

### Total RNA Extraction and Quantitative Real-Time PCR (qRT-PCR)

Cells were suspended in 1 ml TRIzol reagent (15596026, Thermo Fisher Scientific, USA), and RNA was extracted using the RNeasy Micro Kit (74004, QIAGEN, Germany) according to the manufacturer’s instructions and converted to cDNA. mRNA expression was assessed *via* qRT-PCR, performed using a ChamQ Universal SYBR qPCR Master Mix kit (Q711, Vazyme, China).

### Enzyme-Linked Immunosorbent Assay (ELISA)

Supernatants were collected after a 48-h co-cultivation. Cells and apoptotic body free supernatants were concentrated using 3-kDa Amicon Ultra exclusion filters (MilliporeSigma, USA). The concentration of chemokine (C-C motif) ligands (CCL3, CCL4, IL-1β, IL-10, TGF-β) were determined in concentrated supernatants using an ELISA Kit (bioswamp, China), and quantified at 450 nm.

### Flow Cytometry

Macrophages were collected and blocked with 3% bovine serum albumin (BSA) for 45 min, and incubated with anti-human CD86-PE (305406, BioLegend, USA, 1:200), anti-human CD206-APC (321109, BioLegend, USA, 1:200), or anti-human CD68-PE (333808, BioLegend, USA, 1:200) according to the manufacturers’ instructions. Tumor cells were collected and incubated with Annexin V-APC/PI cell death detection kit (abs50001, Absin, China, 1:50) according to the manufacturers’ instructions for 15 min. For each sample, at least 1 × 10^4^ cells were analyzed with the Beckman CytoFLEX flow cytometry. Apoptosis rate (apoptosis%= early apoptotic cells (Upper right quadrant) + late apoptotic cells (Right lower quadrant).

### Western Blot Analysis

Whole cells were lysed in the RIPA lysis buffer supplemented with protease inhibitor cocktail (Roche Diagnostics, USA). Samples were separated using SDS-PAGE (Bio-Rad, USA) and transferred onto nitrocellulose membranes (Bio-Rad, USA). Primary antibodies, including anti-DNMT3A (3598S, Cell Signaling, USA, 1:1000), anti-β-actin (A2066, Sigma-Aldrich, Sweden, 1:1000), as well as horseradish peroxidase-conjugated secondary antibodies (7074, Cell Signaling, USA, 1:1000), and chemiluminescent substrate (Invitrogen, USA) were used.

### Immunofluorescence

Xenografts tissues were fixed with paraformaldehyde, embedded in paraffin, and cut into sections for immunostaining. Mouse monoclonal anti-CD163 (sc-58965, Santa Cruz Biotechnology, USA) and Rabbit anti-CD68 (ab955, Abcam, UK) were used as the primary antibodies. After incubation with the respective primary antibodies in a humidified chamber overnight at 4°C, tissues were rinsed three times with PBS. Then incubated with secondary antibody 1h at room temperature. Finally, the nuclei were counterstained with 4’,6-diamidino-2-phenylindole (DAPI) after washing three times with PBS. After staining, sections were observed under an inverted fluorescence microscope.

### Mouse Xenograft Studies

SCID mice (4 weeks old) were obtained from the Vital River Laboratory Animal Technology Co., Ltd. (Beijing, China). Mice were randomly chosen and divided into five groups (five mice in each group): SKM-1^control^, SKM-1^KO^, SKM-1^NC^, SKM-1^R882H SC1^, and SKM-1^R882H SC2^. AML cells (5 × 10^6^) were mixed with 5 × 10^6^ M1 macrophages and implanted into the armpits of the mice.

### RNA-Sequencing

Total RNA extraction was performed with TRIzol Reagent (Invitrogen, Carlsbad, CA, USA). Extraction of total cell RNA was carried out with the help of RNAiso Plus (TaKaRa, Dalian, China). 1 ~ 2 μg of total RNA per sample (3 SKM-1^WT^ cell samples, 3 SKM-1^KO^ cell samples and 3 SKM-1^R882H SC1^ cell samples) was utilized for constructing a KAPA Stranded RNA-Seq Library Prep Kit (Illumina) sequencing library. Afterwards, the constructed library was cross-checked using an Agilent 2100Bioanalyzer and quantified by qRT-PCR. Lastly, the Illumina HiSeq 4000 (service provided by Kangchen Bio-tech, Shanghai, China) was used to further sequence the libraries that had different mixed samples.

### Statistical Analysis

Data were represented as mean ± SEM. D ‘Agostino and Pearson omnibus normality tests were used to detect the partial normality of experimental data. When the data were not normally distributed, logarithmic transformation was performed to conform to the normal distribution. Differences between groups were tested using one-way ANOVA followed by Sidak test for multiple comparisons or a paired Student t-test as appropriate using Prism 7.0 (GraphPad Software). P ˂ 0.05 was considered statistically significant.

## Results

### Construction of DNMT3A KO Cells and DNMT3A-R882H Mutant Cells

**Figure 1:**
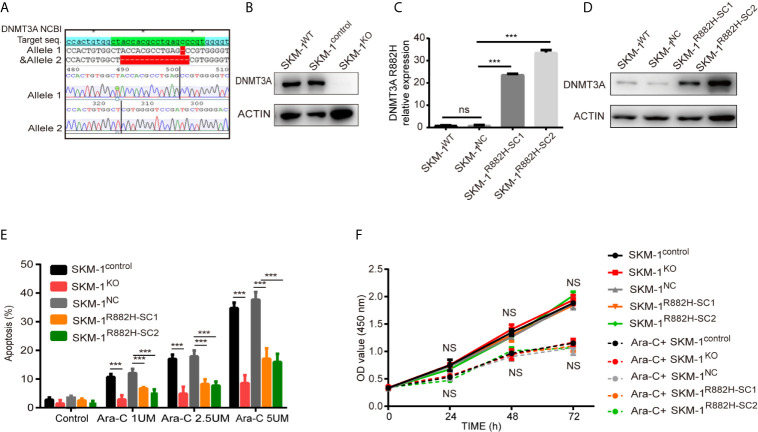
The construction of DNMT3A knockout (KO) cells and DNMT3A-R882H mutant cells. **(A)** -1 and -14 frameshift mutations in the *DNMT3A* allele sequence of SKM-1^KO^ monoclonal cells. **(B)** SKM-1^KO^ cells did not express DNMT3A. **(C, D)** DNMT3A-R882H mRNA and protein expression levels in the constructed SKM-1^R882H SC1^ and SKM-1^R882H SC2^ clones. **(E)** SKM-1^KO^ and SKM-1^R882H^ cells were more resistant to cytarabine than the control group (SKM-1^NC^). **(F)** CCK8 assay showed no significant difference in proliferation of SKM-1^R882H^, SKM-1^KO^, SKM-1^control^, and SKM-1^NC^ cells before and after 1 µM cytarabine treatment. All the results are presented as mean ± standard deviation of three independent experiments (n = 3). ***p < 0.0001 represent significant differences compared with the control. ns, no significance.

CRISPR-Cas9 was used to knock out DNMT3A in wild type SKM-1 cells to obtain SKM-1^KO^ cells. SKM-1^control^ is a monoclonal cell which was generated in the same conditions, but not transfected with sgRNA. DNMT3A-R882H (CGC > CAC) mutant SKM-1 cell strains (SKM-1^R882H^ cells) were constructed *via* lentiviral transfection, and two mutant monoclones (SKM-1^R882H SC1^ and SKM-1^R882H SC2^) were selected. The negative control monoclone (SKM-1^NC^ cells) was transfected with an empty vector. Sanger sequencing showed that SKM-1^KO^ cells had -1 and -14 frameshift mutations in exon 6 of the *DNMT3A* allele (**Figure 1A**). Western blot analysis showed that SKM-1^KO^ cells did not express DNMT3A, and SKM-1^R882H^ mutant strains highly expressed DNMT3A-R882H (**Figures 1B–D**). After cytarabine treatment, the apoptosis rate of the experimental groups (SKM-1^KO^, SKM-1^R882H SC1^, and SKM-1^R882H SC2^ cells) was significantly lower than that of the control groups (wild type SKM-1^control^ cells and SKM-1^NC^ cells) (**Figure 1E**), and the proliferation rate of the experimental groups was not significantly different from that of the control groups (**Figure 1F**). Generation of isogenic K562 clones with the DNMT3A R882H mutation using designed TALENs (19).

### The Difference in Gene Expression Between SKM-1^R882H^, SKM-1^KO^ and SKM-1^WT^ Cells

**Figure 2:**
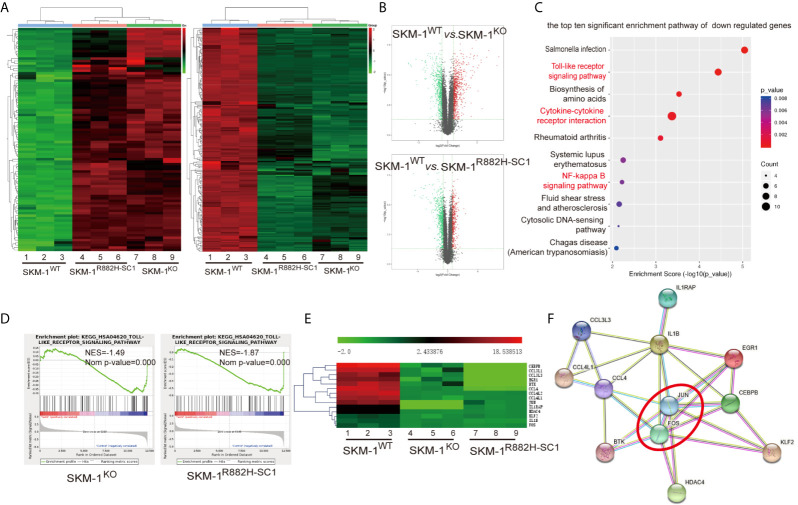
The difference in gene expression between SKM-1^R882H^, SKM-1^KO^ and SKM-1^WT^ cells. **(A)** Cluster analysis of differential genes, red means upregulated expression, green means downregulated expression. **(B)** The volcanic map analysis of differentially expressed genes (fold change ≥ 2 and p-Value ≤ 0.05), the red indicates the significantly upregulated gene, the green indicates the significant downregulated. **(C)** KEGG pathway enrichment analysis. The size of the circle indicates the number of differential genes, and the warm and cold color indicates the size of the P-value. The ordinate indicates the enrichment pathway, and the abscissa indicates the enrichment score. Inflammation- and immune-related pathways was marked in red. **(D)** GSEA indicates that the TLR pathway was downregulated in knockout (KO) and R882H cells. **(E)** Cluster analysis of the differential genes. **(F)** Interaction networks functional enrichment analysis of differential proteins. Red circles indicate key regulatory factors.

RNA-seq results showed that SKM-1^R882H SC1^ and SKM-1^KO^ cells co-expressed 96 upregulated genes and 185 downregulated genes compared with the control group (**Figure 2A**). The overall distribution of differentially expressed genes (fold change ≥ 2 and p-Value ≤ 0.05) was reflected in the volcanic map (**Figure 2B**). Gene set pathway enrichment analysis (GSEA) revealed that the co-expressed differential genes were significantly enriched in inflammation and immune-related pathways, such as NF-κB signaling pathway (P = 0.006) and Toll-like receptor (TLR) signaling pathway (P = 0.00003) (**Figure 2C**). GSEA revealed that the TLR pathway was downregulated in SKM-1^KO^ (NES = -1.49, Nominal p < 0.001) and SKM-1^R882H SC1^ cells (NES = -1.87, Nominal p < 0.001) (**Figure 2D**). Transcription regulators, such as Jun proto-oncogene (JUN), Fos proto-oncogene (FOS), CCAAT enhancer-binding protein β (CEBPB), and Kruppel-like transcription factor 2 (KLF2), as well as inflammatory factors, such as interleukin-1β (IL-1β), macrophage inflammatory protein 1β (MIP-1β or CCL4 (including CCL4, CCL4L1, CCL4L2), and macrophage inflammatory protein 1α (MIP-1α or CCL3, including CCL3L1, CCL3L3), which play an important role in inflammation and immune-related pathways, were significantly downregulated in SKM-1^R882H SC1^ and SKM-1^KO^ cells (**Figure 2E**). We imported these differential genes into the STRING database (https://string-db.org/) for protein-protein interaction network functional enrichment analysis (PPI). The results showed that transcription regulators JUN and FOS were at the center of this interaction network, which indicated that AP-1 (a heterodimer composed of C-FOS and C-JUN) may play a key role in the downregulation of the inflammatory factors (**Figure 2F**). The trend verified *via* qRT-PCR was consistent with that of RNA-seq (data not shown).

### DNMT3A-Mutated AML Cells Attenuated M1 Macrophage Polarization and Resisted Its Killing Effect

**Figure 3:**
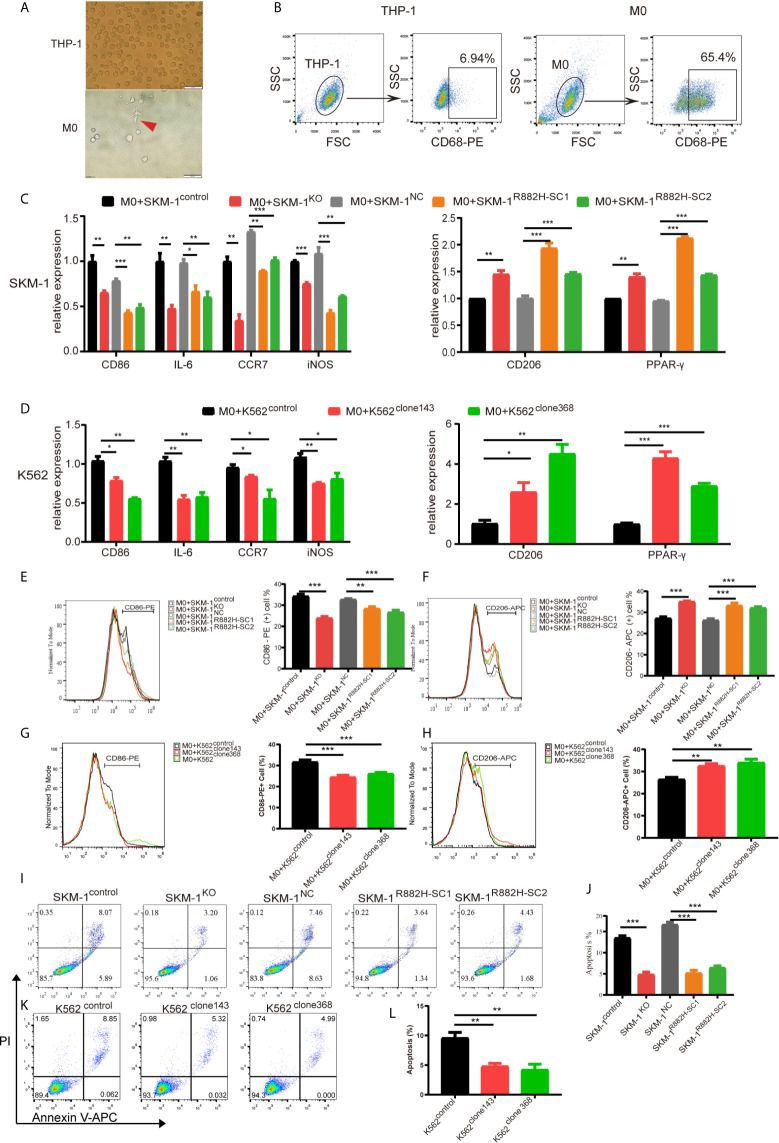
DNMT3A-mutated AML cells attenuated M1 macrophage polarization and resist its killing effect *in vitro*. **(A)** The morphological changes of THP-1 differentiation into M0 macrophages, the red arrow points to M0 macrophages. **(B)** The expression of CD68 in THP-1 after stimulation with PMA. **(C, D)** The mRNA expression of *CD86*, *IL*-*6*, *iNOS*, *CCR7* (M1 marker) and *CD206*, *PPAR*-γ (M2 marker) was measured by qRT-PCR in macrophages after co-cultured with SKM-1 cells and K562 cells respectively. **(E–H)** The expression of CD86 and CD206 in macrophages after co-cultured with AML cells was analyzed using flow cytometry. The histogram is a statistical graph of the flow chart. **(I–L)** Representative dot plots and column diagram showing the apoptosis rate of AML cells after co-cultured with M1 macrophages. All the results are presented as mean ± standard deviation of three independent experiments (n = 3). *p < 0.05, **p < 0.01, and ***p < 0.0001 represent significant differences compared with the control.

Due to the abnormal expression of macrophage-related inflammatory proteins in DNMT3A-mutated cells, we co-cultured AML cells with THP-1-derived macrophages. After PMA induction, THP-1 monocytes became adherent. Under 400× magnification, the cells were polynuclear with antenna formation (**Figure 3A**). The expression of recognized macrophage markers, cluster of differentiation 68 (CD68), was significantly increased (65.4% vs 6.94%, P < 0.001) in THP-1-derived macrophages (**Figure 3B**). These results indicated that THP-1 cells successfully differentiated into macrophages. CD86, IL-6, inducible nitric oxide synthase (iNOS), and CC-chemokine receptor 7 (CCR7) were used as markers of M1 macrophages, while macrophage mannose receptor (CD206), peroxisome proliferator-activated receptor (PPAR-γ) as markers of M2 macrophages. Co-culturing THP-1-derived macrophages and AML cells for 48 h. The results showed that the mRNA expression of M1 macrophage-related markers was significantly downregulated (P < 0.05) in macrophages of the experimental group (SKM-1^KO^ and SKM-1^R882H^ cells, K562^clone143^ and K562^clone368^) (**Figures 3C, D**), while the mRNA expression of M2 macrophage-related markers was significantly increased compared with the control group (SKM-1^control^ cells and SKM-1^NC^ cells, K562^control^ cells) (P < 0.01) (**Figures 3C, D**). Flow cytometry verified the above trends compared with the control group, CD86 expression was downregulated (P < 0.01) and CD206 expression was upregulated (P < 0.01) in macrophages of the experimental group (**Figures 3E–H**). To amplify the killing effect of macrophages on AML cells, we co-cultured AML cells with M1 macrophages. After 48 h, the apoptosis rate of the experimental group was significantly lower than that of the control group (P < 0.05) (**Figures 3I–L**).

**Figure 4:**
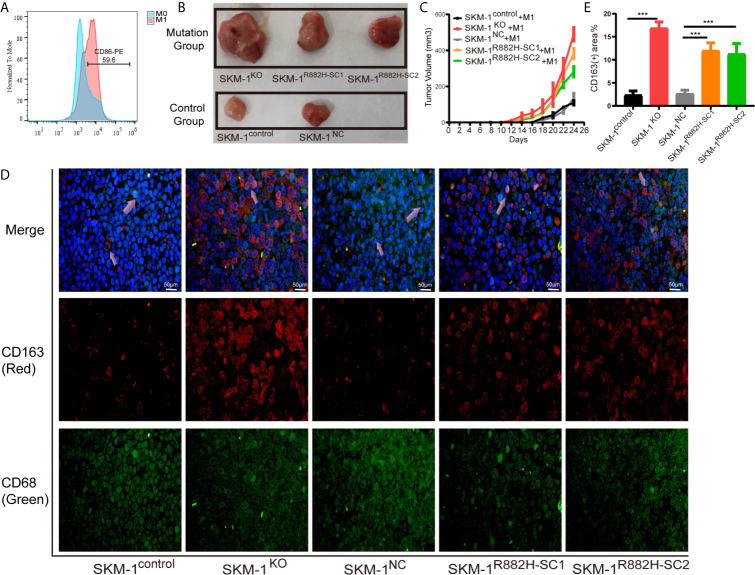
DNMT3A-mutated AML cells attenuated M1 macrophage polarization and resist its killing effect *in vivo*. **(A)** The expression of CD86 in M0 macrophages after stimulation with Lipopolysaccharide (LPS). **(B, C)** The tumor volume of the experimental and the control group after the co-culture with M1 macrophages *in vivo*, n = 3. **(D)** Immunofluorescence showed the number of CD68+CD163+ double-positive cells (M2 macrophages) infiltrating the tumor tissue. CD68 (macrophage marker) in green, CD163 (M2 marker) in red, nuclei in blue, and the purple arrow points to M2 macrophages. **(E)** Statistical graph of CD163-red fluorescence intensity. All the results are presented as mean ± standard deviation of three independent experiments (n = 3). ***p < 0.0001 represent significant differences compared with the control.

We mixed SKM-1 cells with M1 macrophages and injected them subcutaneously into the mice (**Figure 4A**). We found that the growth rate of the experimental group was significantly larger than that of the control group (P < 0.05) (**Figures 4B, C**). Immunofluorescence showed that the proportion of CD68+ and CD163+ double-positive cells (M2 macrophages) infiltrating the tumor tissue of the experimental group was significantly higher than that in the control group (**Figures 4D, E**).

### DNMT3A Mutation Inhibits the Expression of Pro-Inflammatory Factors in AML Cells and Inhibits Anti-Tumor Immunity

**Figure 5:**
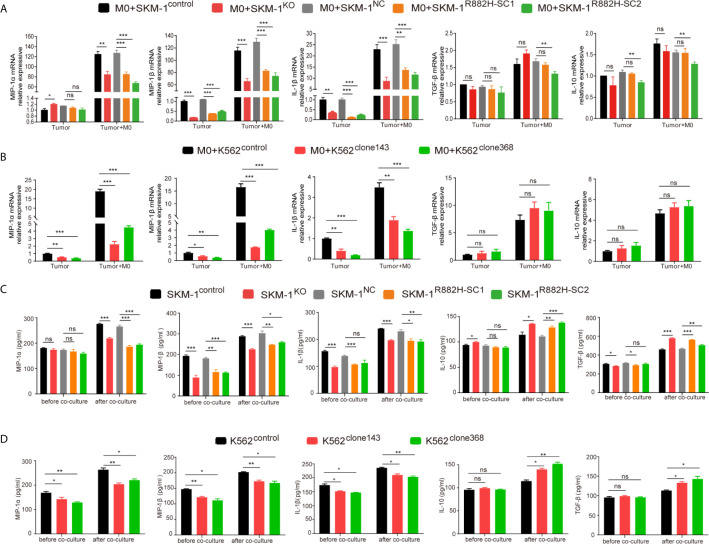
DNMT3A mutation inhibits the expression of pro-inflammatory factors in AML cells and inhibits anti-tumor immunity. **(A, B)** The mRNA expression of MIP-1α, MIP-1β, IL-1β (pro-inflammatory factors) and TGF-β, IL-10 (immunosuppressive factors) was measured by qRT-PCR in SKM-1 cells and K562 cells respectively after co-cultured with macrophages. **(C, D)** The secretion of inflammatory factors in the co-cultured supernatant was measured by ELISA. All the results are presented as mean ± standard deviation of three independent experiments (n = 3). *p < 0.05, **p < 0.01, and ***p < 0.0001 represent significant differences compared with the control. ns, no significance.

Co-cultivation promoted the expression of the inflammatory factors in AML cells. However, the rise in the expression of MIP-1α, MIP-1β and IL-1β in the experimental group was significantly lower than that in the control group (P < 0.01), while IL-10 and TGF-β expression in the experimental group was not significantly different from that in the control group (P > 0.05) (**Figures 5A, B**).

In the supernatant of the co-culture system, the concentration of inflammatory factors MIP-1α, MIP-1β, and IL-1β in the experimental group was significantly lower than that in the control group, as revealed using ELISA (P < 0.05). Moreover, the concentration of immunosuppressive factors IL-10 and TGF-β was significantly higher than that of the control group (P<0.05). This indicated that in the experimental group microenvironment, anti-tumor immunity was suppressed compared with the control group (**Figures 5C, D**).

### DNMT3A-Mutated AML Cells Regulate the Macrophage Phenotype by Suppressing the Transcription Factor AP-1

**Figure 6:**
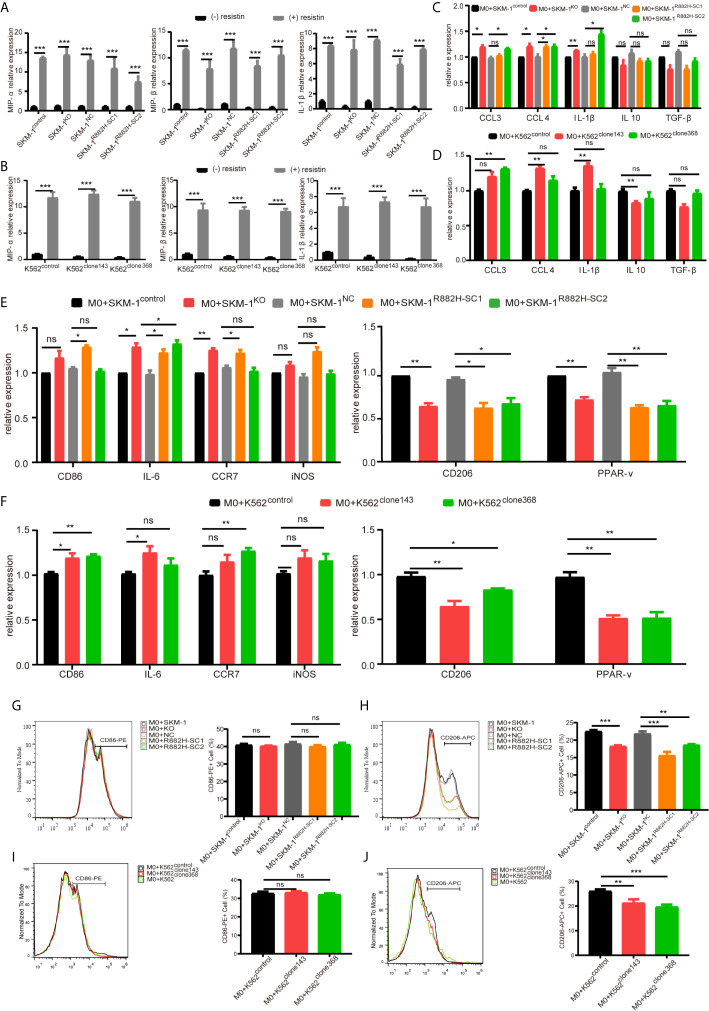
DNMT3A mutated AML cells regulate the macrophage phenotype by suppressing transcription factors AP-1. **(A, B)** The mRNA expression of *MIP-1α*, *MIP-1β*, *IL-1β* was measured by qRT-PCR in SKM-1 cells and K562 cells after stimulation with resistin. **(C, D)** The mRNA expression of *MIP-1α*, *MIP-1β*, *IL-1β* (pro-inflammatory factors) and *TGF-β*, *IL-10* (immunosuppressive factors) was measured using qRT-PCR in SKM-1 cells and K562 cells treated with resistin after co-cultured with macrophages. **(E, F)** The mRNA expression of *CD86*, *IL-6*, *iNOS*, *CCR7* (M1 marker) and *CD206*, *PPAR-γ* (M2 marker) was measured using qRT-PCR in macrophages after the co-culture with AML cells treated with resistin. **(G–J)** The expression of CD86 and CD206 in macrophages after the co-culture with AML cells treated with resistin was analyzed using flow cytometry. The histogram is a statistical graph of the flow chart. *p < 0.05, **p < 0.01, and ***p < 0.0001. ns, no significance.

**Figure 7:**
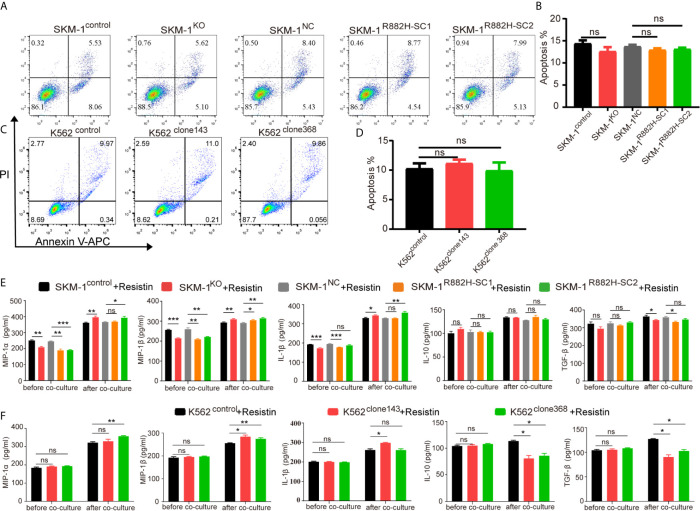
DNMT3A mutated AML cells regulate the macrophage phenotype by suppressing transcription factors AP-1. **(A–D)** Representative dot plots and column diagram showing the apoptosis rate of AML cells treated with resistin after the co-culture with M1 macrophages. **(E, F)** The secretion of inflammatory factors in the supernatant of macrophages and resistin-treated AML cells was measured by ELISA. All the results are presented as mean ± standard deviation of three independent experiments (n = 3). *p < 0.05, **p < 0.01, and ***p < 0.0001 represent significant differences compared with the control. ns, no significance.

AP-1, an intracellular transcriptional activator, is a heterodimer composed of C-FOS and C-JUN. Considering that AP-1 is at the center of our protein-protein interaction network, we selected resistin, an AP-1 agonist, to verify the key regulatory role of AP-1. The results showed that resistin significantly increased the expression of MIP-1α, MIP-1β, and IL-1β in AML cells (P < 0.001) (nearly ten times) (**Figures 6A, B**). After treating AML cells with resistin for 12 h, we co-cultured them with macrophages. It narrowed the difference in the expression levels of MIP-1α, MIP-1β, and IL-1β between mutant and control groups after the co-culture, and even promoted the mutant group to further express MIP-1α, MIP-1β and IL-1β (**Figures 6C, D**). Still, there was no significant difference in the expression of anti-inflammatory factors IL-10 and TGF-β between mutant and control groups (**Figures 6C, D**). As to the co-cultured macrophages, the results showed that the mRNA expression of M1 macrophage-related markers in the mutant group was not lower than that in the control group, while the mRNA expression of M2 macrophage-related markers was significantly decreased compared with that in the control groups (P < 0.05) (**Figures 6E, F**). Flow cytometry verified the above trends; CD86 expression in the mutant group was not significantly different from that in the control group, and CD206 expression was downregulated (P < 0.01) in macrophages of the experimental group (**Figures 6G–J**). We also co-cultured the resistin-treated AML cells with M1 macrophages for 48 h. There was no significant difference between the apoptosis rate of the mutant groups and that of the control group (**Figures 7A–D**). We detected the concentration of inflammatory factors in the supernatant of macrophages and AML cells treated with drugs. The results showed that after co-cultivation the concentration of pro-inflammatory factors MIP-1α, MIP-1β, and IL-1β in the supernatant of the experimental group was no longer lower than that in the control group (even slightly higher), and the concentration of immunosuppressive factors IL-10 and TGF-β was no longer higher than that in the control group (or even slightly lower) (**Figures 7E, F**).

## Discussion

DNMT3A reportedly undergoes an R882H mutation in approximately 25% of patients with M4/M5 subtype AML (3). Although DNMT3A mutations affect many different sites in the coding region, Arg882 (R882) mutation within the catalytic domain of DNMT3A is the most common type (20, 21). Therefore, we constructed DNMT3A-R882H mutated cells in SKM-1 cell line using lentiviral transfection and K562 cell line using designed TALENs. Mutations in DNMT3A cause loss of enzyme activity (22, 23). In vitro experiments showed that murine DNMT3A with the R878H mutation, which corresponds to human DNMT3A R882H, fails to mediate DNA methylation, suggesting the dominant-negative role of the DNMT3A R882H mutation (24). To amplify the inactivation of mutated DNMT3A protease, we constructed SKM-1^KO^ cells using CRISPR-Cas9 technology. Although the difference in proliferation was not obvious, we discovered that mutant clones exhibited significantly promoted chemo-resistance, which was consistent with the previous studies (25). These results indicated that we successfully constructed DNMT3A mutant clones.

As one of the most important epigenetic modifications, DNA methylation is considered to be an important factor for inflammatory response and immune cell differentiation (26). Previous studies showed that DNMTs promote the expression of genes involved in inflammation, such as *IL-6*, *TNF-α*, *IL-1β*, *NO*, and *CCL2* (27). Our RNA-seq data revealed that transcription regulators (JUN, FOS, CEBPB, KLF2) and inflammatory factors (IL-1β and MIP-1 and their truncated proteins) were downregulated in DNMT3A mutant clones. The truncated proteins CCL3L and CCL4L are protein hydrolysates of MIP-1. They can improve the biological activity of MIP-1 (28); therefore, in DNMT3A mutant AML cells, the expression and activity of MIP-1 are significantly downregulated. As for transcription regulators, the homodimers and heterodimers of JUN and FOS regulate gene transcription by forming the activator protein-1 (AP-1) (29). Studies have shown that AP-1 can regulate the expression of MIP-1α, MIP-1β, and IL-1β by specifically binding to the promoter region in inflammation and tumors (30, 31). Inflammation signaling pathways, such as TLR pathway, can directly regulate the expression of AP-1 (32–34). In the present study, the co-expressed differential genes were significantly enriched in inflammation- and immune-related pathways, such as the NF-κB signaling pathway and TLR pathways. They play an important role in the inflammatory response and macrophage polarization. A growing body of evidence showed that the two signaling pathways can both be regulated through DNA methylation (35, 36).

Although DNMT3A mutation is associated with poor prognosis in AML, the mechanism is unclear (21). Studies have shown that DNMT3A mutation can prevent the differentiation of hematopoietic stem cells and leukemia cells *via* polycomb repressive complex 1 (37), and promote resistance to chemotherapy by interfering with the glutathione metabolism (19). DNMT3A mutation in mice promotes the development of leukemia by upregulating CDK1 through the mTOR pathway (38). However, there are few reports on the relationship between DNMT3A-mutated AML and immune microenvironment. Considering the abnormal expression of macrophage-related inflammatory proteins in DNMT3A-mutated cells, we co-cultured AML cells with THP-1-derived macrophages. We found that the polarization of macrophages co-cultured with the mutant group was significantly different from that of the control group. Furthermore, DNMT3A-mutated AML clones resist the killing effect exerted by M1 macrophages *in vitro* and *in vivo*. AML cells have a stronger ability to chemoattract M2 macrophages than normal cells (39). Our data showed that DNMT3A mutation enhanced this ability of AML cells. Moreover, researchers found that in the DNMT3A-R882H mutated mice, the growth of monocytic/megakaryocytic lineages has obvious growth advantages (40). This indicates that AML with DNMT3A mutation has various effects on macrophages. In addition, the immunophenotype of DNMT3A-mutated AML cells is chemotactic to the mononuclear line (41), and researchers found that the differentiated monocyte-like AML cells inhibit T cell activity (42). These results indicate that AML with DNMT3A mutation may affect a variety of immune cells in the microenvironment. The proportion of M2 macrophages in the bone marrow of patients with AML is significantly higher than that of healthy donors and patients with other types of leukemia (39). High expression of M2 macrophage markers, such as CD163 and CD206, is significantly correlated with short survival time (39, 43). This may explain to a certain extent why the prognosis of AML with DNMT3A mutation is worse.

In conclusion, our data revealed that the DNMT3A-mutated AML cells attenuate M1 macrophage polarization and resist its killing effect *in vitro* and *in vivo*. Furthermore, our results indicated that the mechanism is related to DNMT3A inhibiting the expression of inflammatory factors MIP-1α, MIP-1β, and IL-1β through the AP-1 site. As resistin can reverse this trend, it may serve as an ancillary drug for patients with DNMT3A-mutated AML. In summary, our study showed that DNMT3A-mutated AML cells achieve immune escape by regulating the phenotype of macrophages. Our study provides a novel explanation for the underlying mechanism of the poor prognosis of DNMT3A-mutated AML.

## Data Availability Statement

The raw data supporting the conclusions of this article will be made available by the authors, without undue reservation.

## Ethics Statement

The animal study was reviewed and approved by Laboratory Animal Welfare Ethics Committee of Tongji Hospital Affiliated to Tongji Medical College of Huazhong University of Science and Technology.

## Author Contributions

HL, YQ, and DL conceptualized this study. YQ and HL completed data curation. LL, XZ, MX, LZ, and YW conducted the investigation. YQ, HL, LL, XZ, MX, LZ, YW, and DL completed the methodology. DL was responsible for project administration. XZ and MX were in charge of software. YQ and HL wrote the original draft. DL wrote, reviewed, and edited the manuscript. All authors contributed to the article and approved the submitted version.

## Conflict of Interest

The authors declare that the research was conducted in the absence of any commercial or financial relationships that could be construed as a potential conflict of interest.
