# Supplementary material for: Study on the Immune Escape Mechanism of Acute Myeloid Leukemia With DNMT3A Mutation
Source: Front Immunol. 2021 May 20;12:653030. doi: 10.3389/fimmu.2021.653030 (PMC8173207; doi:10.3389/fimmu.2021.653030)
Supplement: Supplementary file 1 [file DataSheet_1.docx]

**Supplementary Appendix**

This appendix is provided by the authors for additional information about their work.

Supplements to Yimei Que*, et al.* Study on the immune escape mechanism of acute myeloid leukemia with DNMT3A mutation

**SUPPLEMENTAL MATERIALS**

**CONTENTS:**

**Supplementary figures**

Figure S1. The apoptosis rate of SKM-1^control^, SKM-1^KO^, SKM-1^NC^, SKM-1^R882H SC1^ and SKM-1^R882H SC2^ treated with different concentration of cytarabine.

Figure S2. DNMT3A mRNA expression levels in SKM-1^WT^, SKM-1^control^ and SKM-1^KO^ clones.

Figure S3. The time gradients and E: T gradients in vitro co-culture.

Figure S4. The expression of CD86 and CD206 in macrophages after co-cultured with SKM-1^control^, SKM-1^KO^, SKM-1^NC^, SKM-1^R882H SC1^ and SKM-1^R882H SC2^.


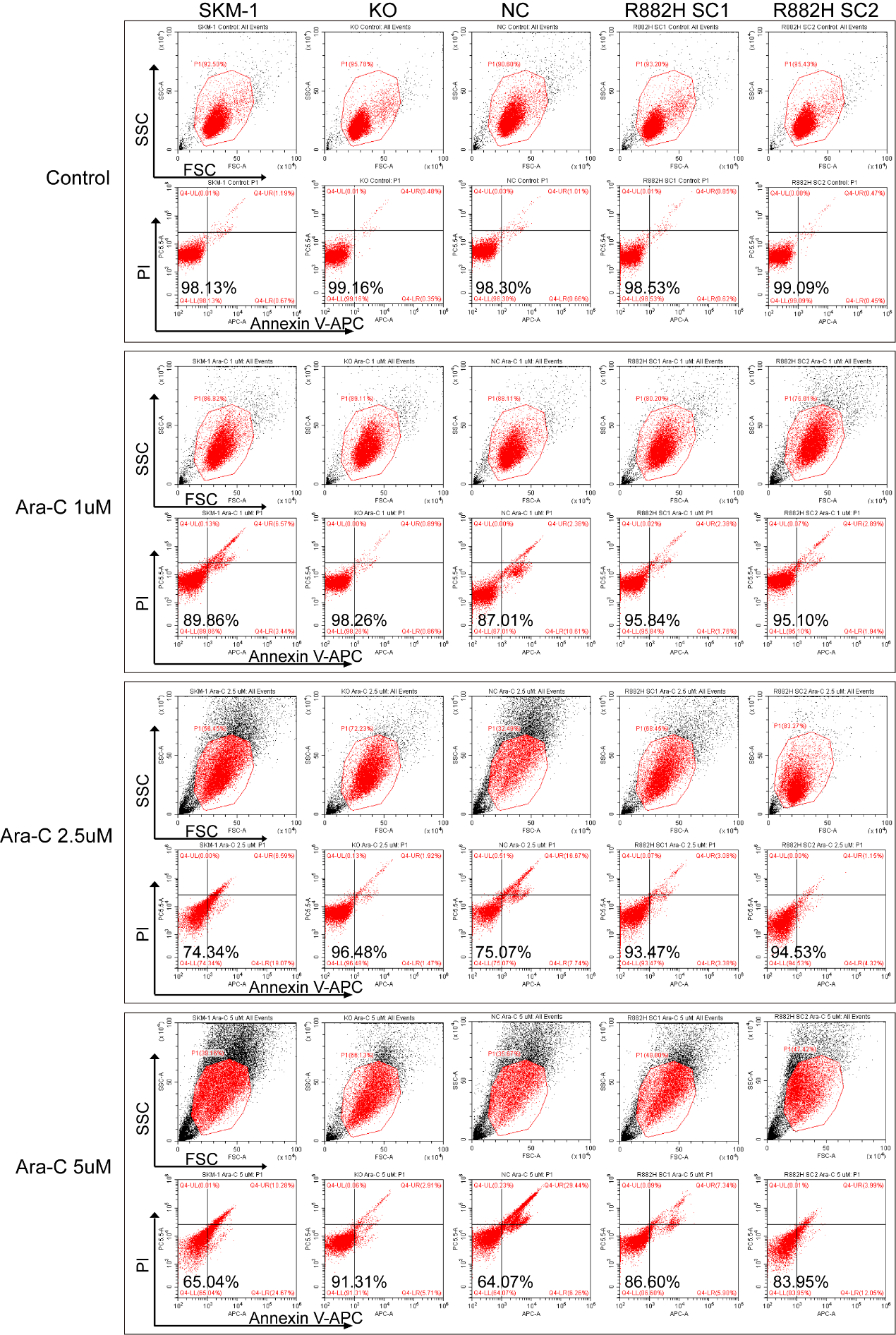


**Figure S1. The apoptosis rate of SKM-1^control^, SKM-1^KO^, SKM-1^NC^, SKM-1^R882H SC1^ and SKM-1^R882H SC2^ treated with different concentration of cytarabine.** SKM-1^KO^, SKM-1^R882H SC1^ and SKM-1^R882H SC2^ cells were more resistant to cytarabine than the control group (SKM-1^control^ and SKM-1^NC^).


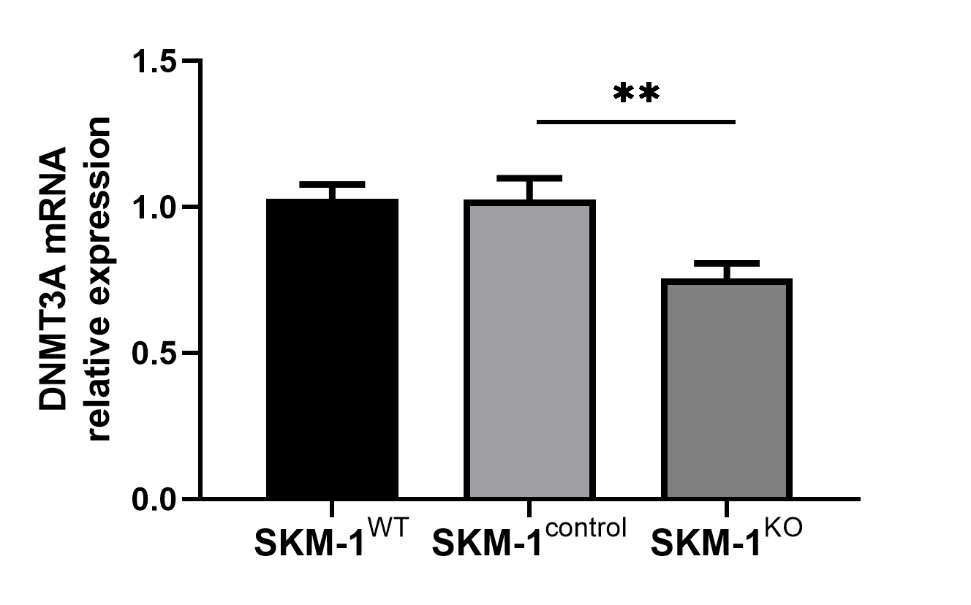


**Figure S2. DNMT3A mRNA expression in SKM-1^WT^, SKM-1^control^ and SKM-1^KO^ clones.** There was no significant difference of DNMT3A mRNA expression between SKM-1^WT^ clone and SKM-1^control^ clone. DNMT3A mRNA was slightly down-regulated in SKM-1^KO^ clone.


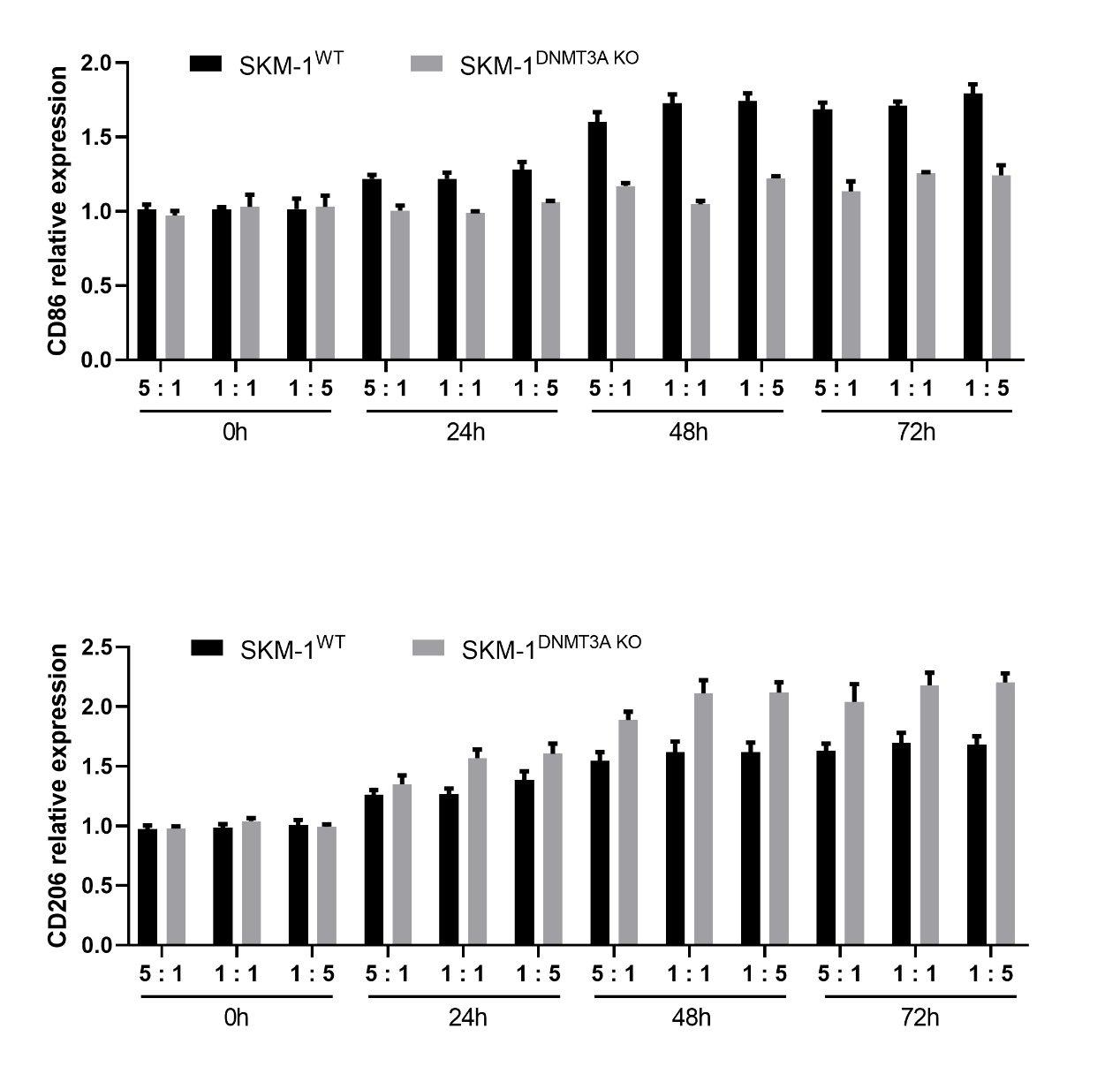


**Figure S3. The time gradients and E: T gradients in vitro co-culture.** SKM-1 cells were co-cultured with macrophages. The expression of CD86 and CD206 in macrophage increased over time. E:T ratio (macrophages: SKM-1 cells) had no significant effect on the result.


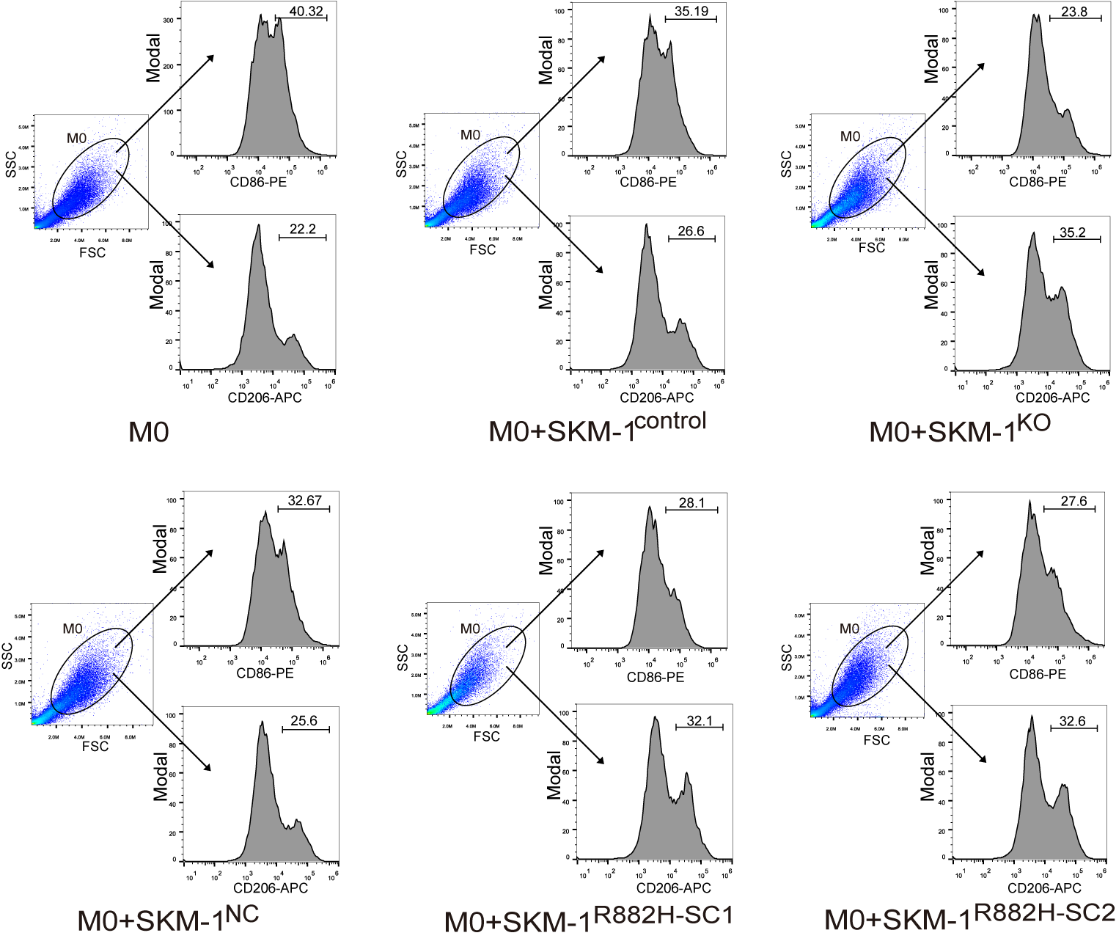


**Figure S4. The expression of CD86 and CD206 in macrophages after co-cultured with AML cells was analyzed using flow cytometry.** There is the raw data of gating strategies and M0 controls of Figure 3E-F.
